# A Short Synopsis of the Last Annual Meeting of the Military Tract Medical Association

**Published:** 1874-08-01

**Authors:** 


### A SHORT SYNOPSIS OF THE LAST ANNUAL MEETING OF THE MILITARY TRACT MEDICAL ASSOCIATION.

THE 8th annual meeting of the Military Tract Medical Association convened pursuant to adjournment at Kewanee, July 14th. The meeting was held in the Presbyterian church. Although hardly the usual number of members were present, the reports from the several committees were full, and the time did not permit all the papers to be read. The valedictory address of M. A. McClelland, of Knoxville, the retiring President, "Medicine—Past and Present," was distributed among the Association in pamphlet form. Wm. Hamilton, of Galesburg, was elected President for the ensuing year; A. C. Babcock, of Galva, and J. F. McCutcheon, of Norwood, as first and second Vice Presidents; Herbert Judd, of Galesburg, as Secretary and Treasurer. Dr. Nance, of Kewanee, presented several cases under surgical treatment, and also read a paper upon skin diseases. This paper was accepted with a vote of thanks from the Association, and referred for publication.

Dr. McCutcheon gave a written and verbal report of cases under treatment. For the want of time this report did not receive the discussion it seemed to require.

Dr. Scott, of Galesburg, sent a report as Chairman of the Committee on Obstetrics and Diseases of Women. This paper, read by the Secretary, was received by a vote of thanks. Accompanying Dr. Scott's paper was a report sent him of the Scalf case, prepared by Dr. J. T. Stewart, of Peoria. This report was very much to the favor of Dr. Lucas, the attending physician in this case, and was also written in a manner to criminate Dr. Skinner, of Peoria, the consulting physician in this case. The paper from Dr. Stewart was read by the Secretary, and as the members present wished to see fair play, Dr. Skinner, who was present, was requested to give his statement of the case, at the close of which, after a lengthy discussion, the following resolution was adopted:

WHEREAS, A report on Obstetrics and Diseases of Women has been sent into our Society, to be read by one of our number, and at the close of said report an attempt has been made to make public a case not within the jurisdiction of our Society relating to this branch of medicine; therefore, We, the members of the Military Tract Medical Association, assembled at Kewanee, this 14th day of July, 1874, do most seriously deplore the course taken by a number of our profession of Peoria in sending such statements to the Chairman of Committee on Obstetrics, and we hereby request of said Chairman an explanation of the reasons which prompted his action in the premises.

Prof. A. D. Williams, of St. Louis, who had been invited to be present by Dr. L. S. Lambert, of Galesburg, was made an honorary member of the Association. Also, at the request of the Association, Prof. Williams gave a short report from his branch of the profession, and also gave the history of a few cases. This report was received by the Association with a vote of thanks and referred for publication.

Other reports which had been prepared were not presented, for want of time.

After the appointment of the several committees to report at the January meeting, and the usual miscellaneous business, the Association adjourned, to meet at Galva on the second Tuesday in January next.

At the next meeting of this Association action will be taken to have but one meeting each year, with two or more days session. This Association has over one hundred active members, and one day is too short a time to meet the present requirements.

